# Benefits of Kangaroo Mother Care on the Physiological Stress Parameters of Preterm Infants and Mothers in Neonatal Intensive Care

**DOI:** 10.3390/ijerph19127183

**Published:** 2022-06-11

**Authors:** Delia Cristóbal Cañadas, Tesifón Parrón Carreño, Cristina Sánchez Borja, Antonio Bonillo Perales

**Affiliations:** 1 Neonatal and Paediatric Intensive Care Unit, Torrecárdenas University Hospital, 04009 Almería, Spain; 2 Department of Nursing, Physiotherapy and Medicine, University of Almería, 04120 Almería, Spain; tpc468@ual.es; 3 Andalusian Council of Health at Almería Province, 04005 Almería, Spain; 4 Pediatrics Department, Torrecárdenas University Hospital, 04009 Almería, Spain; csborja.89@gmail.com (C.S.B.); abonillop@gmail.com (A.B.P.)

**Keywords:** kangaroo mother care, preterm, stress, cortisol, neonatal intensive care

## Abstract

It is well documented that the stress of separation of mother and baby can lead to short-term physiological instability as well as neurological, sociological or psychological consequences that may last a lifetime. Objective: The goal was to estimate the effect of kangaroo mother care (KMC) on physiological and biochemical parameters of preterm infant stress and maternal stress in neonatal intensive care. Methods: The investigation involved 112 preterm infants. Two groups were compared according to the mean duration of KMC during 12 days of study: the KMC group (mean duration more than 90 min daily) and the control group (less than 90 min). Results: Kangaroo mother care for more than 90 min on average per day in preterm infants is associated 12 days after the intervention with lower mean cortisol levels (*p* = 0.02), greater weight gain and less need for parenteral nutrition in preterm infants, as well as less postpartum depression (*p* = 0.02) and lower cortisol levels (*p* = 0.002) in the mothers of preterm infants. Conclusions: This study suggests that KMC can be used to improve the stress of preterm infants and their mothers, and that the greater weight gain observed in these preterm infants could contribute to a shorter average hospital stay and lower healthcare expenditure.

## 1. Introduction

Preterm birth is a major global health problem. Low birth weight is a major contributor to both neonatal and infant mortality [1]. Every year, 15 million babies are born preterm, and this number is increasing [2]. Around 30,000 premature babies are born in Spain every year. [3,4]. Of these, 10% have a gestational age (GA) ≤ 32 weeks or a birth weight (BW) ≤ 1500 g. The lower the birth weight and gestational age of preterm infants, the greater their susceptibility to complications resulting from prematurity [5]. Infants in neonatal intensive care units (NICUs) are subjected to numerous stressors, such as excessive noise and light levels [6,7], frequent medical or nursing interventions and problems related to separation from the mother such as limited visual, acoustic and tactile interactions between mothers and babies [8,9], which can affect neurodevelopmental outcomes in infants born preterm [5]. In turn, these limited interactions provoke anxiety in mothers, affect maternal bonding and exacerbating with the stressful experience of having a preterm baby [8].

Stress activates the hypothalamus–pituitary–adrenocortical (HPA) axis, triggering a complex feedback loop involving the interaction among the hypothalamus, pituitary and adrenal glands, leading to the secretion of the glucocorticoid cortisol, which is considered an indicator of stress as its plasma levels are elevated due to mental and physical stress reactions [10]. Various studies that have used cortisol as an indicator of HPA system activity show that early developmental experience influences the long-term response of the HPA system to stress [11,12,13]. In light of the above and considering the importance of cortisol in the regulation of behaviour, cognition and vulnerability of preterm infants to neurodevelopmental problems, it is essential to gain a better understanding of the mechanisms underlying changes in the HPA axis function [14,15]. In addition, elevated cortisol levels can produce side effects such as insulin resistance, hyperlipidaemia, hyposecretion of growth hormones and changes in the hippocampus, and these high levels are associated with behavioural and somatic disorders [12,16,17]. Cortisol is usually determined in newborns in serum and saliva. Serum cortisol is highly correlated with salivary cortisol measured in preterm infants [18]; however, there are few studies in the literature examining stress regulation in preterm infants measured by salivary cortisol levels that address the kangaroo method. Salivary cortisol has the limitation of having to require a sufficient amount of saliva for analysis due to the peculiar hyposalivation of preterm infants [19] despite the use of a stimulant [20]. Some studies have focused on maternal separation, examining stress and neuroendocrine responses to separation [21], but only a few studies have focused on early separation of preterm infants and the importance of maternal contact in the first few days and weeks of life [22]. Studies suggest that skin-to-skin contact may be a way to reduce stress in infants and is associated with a significant premature reduction in cortisol levels [10,11,12,14,17,21,22]. Indeed, several studies have shown how touch and feel with the babies are associated with HPA axis activity, particularly during critical periods of development [23], but more studies are needed on the association between cortisol and mother–infant interactions in preterm infants.

The practice of kangaroo care has recently begun to gain increased attention and importance in hospitals around the world. The World Health Organisation (WHO) defines Kangaroo Mother Care (KMC) as “the care of premature babies carried skin-to-skin with the mother. It is a powerful and easy-to-use method to promote the health and well-being of infants born preterm and full-term” [24]. The definition includes as key characteristics “early, continuous and prolonged skin-to-skin contact between mother and baby, exclusive breastfeeding (ideally) and early discharge from hospital” [24]. Kangaroo care was developed in Bogotá in the 1970s, where the advantages of early mother–baby contact were recognised, when only a few incubators were available to care for low-birth-weight babies. The WHO in 2003 recognised kangaroo mother care as the most effective way to maintain body temperature, stimulate the senses and provide maternal love. In low-income settings, the original method is applied with 24 h/day skin-to-skin care called continuous KMC. In more affluent settings, the method is applied in limited skin-to-skin sessions, for example one or a few hours, not necessarily every day, occurring over a limited period, called intermittent KMC. Intermittent kangaroo care is used in NICUs, as the kangaroo mother intervention is offered to preterm and/or low-birth-weight infants as long as the baby can tolerate it [25]. In infants, especially preterm infants, stress has been shown to have potentially long-lasting effects on brain organisation and neuroendocrine responses to stress. Furthermore, epigenetic changes have been reported in preterm infants exposed to high levels of stress in the neonatal period [26,27], such as deoxyribonucleic acid (DNA) methylation and histone changes that play a role in normal development [28]. Kangaroo care is an intervention that can influence stress and attachment in mothers of preterm infants [29] and also enhances infant–mother interaction, bonding and attachment, essential for emotional and social development [30]. However, the optimal duration of kangaroo care necessary to optimise its beneficial effects as well as for breastfeeding has not yet been determined [11,31,32].

As mentioned above, preterm birth can be stressful and traumatic for mothers and can have negative consequences for the natural establishment of the mother–infant relationship, affecting the mother’s psychological state, bonding and interactions with her baby [33]. The NICU environment disrupts the mother’s involvement in infant care and jeopardises the attachment process between parents and baby. Mothers play an important role in infant care, and positive interactions between mother and preterm infant at an early age have been shown to be associated with better cognitive outcomes [32,34], and thus maternal involvement appears to have a very positive effect [35]. A growing body of research has shown that parents experience extreme distress, anxiety and depression during their children’s hospitalisations in these units, and symptoms of stress disorder persist after discharge [36,37]. Disruption of their parental role is recognised as the most important stressor for parents of preterm infants [38,39]. Srinath et al. [40] compared the physiological and biochemical responses of stable preterm infants and their parents after KMC and kangaroo father care (KFC), finding no significant differences in physiological responses and stress; however, a recent meta-analysis of parental stress in the NICU concluded that mothers reported higher levels of stress than fathers partly because mothers often play the role of primary caregivers and tend to spend more time than fathers in the NICU, and the constraints to breastfeeding are much more distressing for mothers than for their partners. [38]. However, by providing skin-to-skin contact, mothers see themselves as an important part of the baby’s care through their physical closeness, and this seems to alleviate negative feelings through their involvement in the care of their baby [29].

Although researchers over the last decade have called for increased care and support for both preterm infants and their mothers in NICUs, including interventions such as kangaroo care [6,41], there is considerable variation in practice among different NICUs. This may be related, in part, to concerns about the frailty and haemodynamic instability of preterm babies and concerns about the safety of transferring babies to their mothers in the presence of the necessary tubes and catheters [42], cultural and/or facility and furniture issues [43,44].

Moreover, to date, there is no consensus on the duration of kangaroo mother care that is necessary to optimise beneficial effects and stress reduction [25,26]. Therefore, and based on previous research with preterm infants [21,45,46], we decided to study whether kangaroo care would positively influence serum cortisol levels, an indicator of stress in preterm infants as well as in mothers due to skin-to-skin effects that promote infant stress regulation in neonatal intensive care. The study aimed to estimate the efficacy of prolonged kangaroo care in very preterm infants and its influence on neonatal and maternal stress and the results on physiological stress parameters. The present study is the first to evaluate the effects of KMC and its impact on blood biochemical stress (cortisol), physiological parameters and associated factors in preterm infants at the gestational age of 28–34 weeks and their mothers. 

## 2. Materials and Methods

### 2.1. Study Design and Participants

This cohort study of preterm infants was conducted on 112 preterm infants with a gestational age of 28–34 weeks. Inclusion criteria: Mothers with stable preterm infants born at 28–34 weeks gestational age and admitted to the NICU of the Torrecárdenas University Hospital (Spain) for care were eligible to participate in the present study. Neonates with major congenital anomalies, perinatal asphyxia and intraventricular haemorrhage grades 3 or 4, periventricular leukomalacia, hydrocephalus, encephalopathy, genetic malformations and chromosomal syndromes and those who had received corticosteroid treatment were excluded. A total of 124 preterm infants were assessed for eligibility. Twelve infants were excluded: 6 had haemodynamic instability, and 6 mothers refused to participate, so the final sample was 112 preterm infants. The mothers of the newborns gave their written consent before participating in the study. The study was conducted in accordance with the Declaration of Helsinki, and the protocol was approved by the Ethics Committee of Torrecárdenas University Hospital (PI.DCC/MMC-2019).

### 2.2. Procedures

Recruitment took place from May 2019 to December 2021. Parents were informed of the study by research staff, if eligible, gave consent and completed baseline questionnaires. Included infants started the study from the third day of life, once stabilised, and were studied for the following 12 days, with hospital course data collected daily from the unit’s health record. All preterm infants were able to perform kangaroo care. Those preterm infants whose mothers wanted to perform kangaroo care received a KMC session of at least 90 min per day; this average time was taken as a reference since the WHO [24] advises avoiding kangaroo sessions of less than 60 min, and a systematic review and other recent articles with studies estimate times between 60 and 120 min for the method to offer advantages [11,21,47]. Subsequently, they were then divided into two groups: those who received an average of more than 90 min of KMC per day during the 12 days of the study (*n* = 56) were considered the “kangaroo care group”, and the “control group” was made of up those who received an average of less than 90 min of KMC per day during the 12 days (*n* = 56) “. The duration of each KMC session was determined by the condition of the infants and the availability of the parents.

The data collection tool was specifically designed prior to the start of the study. The tool incorporates a series of scales for the measurement of outcome variables, physiological and anthropometric parameters and demographic information on the mother’s child (age, sex, type of delivery, gestational age, birth weight, body weight at the time of the study, etc.). Weight for the gestational age was classified according to Intergrowth-21 [48] z-scores considering weight as low for the gestational age if the birth weight was below the 10th percentile, normal weight if it was between the 10th and 90th percentiles and high for the gestational age if it was above the 90th percentile. The variables analysed were neonatal physiological stress parameters, neonatal and maternal cortisol levels as a biochemical marker of stress and associated variables such as weight gain and maternal depression. Mean physiological parameters (respiratory rate, heart rate, blood pressure and oxygen saturation) and infant and maternal cortisol, associated variables and the Edinburgh Depression Scale (EDPS) were recorded, first in a baseline measurement (day three) on the day the kangaroo mother programme started and a second one 12 days after the start of KMC (the mean of four daily measurements every 6 h was taken). A triple-blinded analysis was performed with the blinding of participants, data collectors and laboratory staff and evaluators. All infants received standard neonatal care according to NICU protocols and were clinically monitored during the study days.

*Kangaroo mother care*. During the kangaroo session, babies (swaddled only) were placed in an upright position, head turned to one side, legs and arms bent, in direct skin-to-skin contact with the mother, usually with the mother’s breast exposed. Mothers were reclined in a chair with a blanket over the breasts. The duration of the kangaroo session was at least 90 min per day. Whether kangaroo care was performed or not, all preterm infants received routine neonatal care according to the NICU protocol. Parents were able to visit their babies at any time of the day.

*Cortisol sampling*. A blood sample was taken from each infant between 8 and 9 am to minimise the effects of circadian rhythm, with cortisol being analysed from the blood sample taken from routine NICU blood tests to avoid another painful procedure. Cortisol concentrations were quantitatively assessed using an electrochemiluminescence immunoassay. No painful intervention was performed at least 8 h before sampling.

*Postpartum depression* was assessed using the Edinburgh Postnatal Depression Scale (EPDS) [49]. The EPDS is a 10-item self-report questionnaire for the detection of postpartum depression symptoms. The total score is obtained by adding the scores of each of the 10 items. In this study, the Spanish version of the EPDS scale [50] was used, which showed good internal consistency (Cronbach’s α of 0.85). The maximum score on the scale is 30 and the minimum is 0, with higher scores indicating more depressive symptoms. In the analysis, EPDS scores were used as continuous variables (based on total test score) and categorical variables (a score of 10 or more was used to indicate probable major postpartum depression) [51].

### 2.3. Sample Size

The sample size needed to conduct the study was calculated using Epi Info 4.2, estimating to detect a mean difference of 1.8, a common standard deviation of 3.3, a confidence level of 95% and a power of 80%. The result shows that a total of 54 participants were needed in each of the groups, for a total size of 108 participants. The study analysed 112 children and their mothers (56 in the experimental group and 56 in the control group).

### 2.4. Statistical Analysis

Data were analysed using the statistical package SPSS (IBM SPSS, Armonk, NY, USA) version 27.0 and G*Power 3.1.9.7 (software’s company, Aichach, Alemania). Frequencies and percentages were used for qualitative variables and means and standard deviation for quantitative variables. The Chi-squared test (χ^2^) was used to compare qualitative variables. For the comparison of quantitative variable averages, Student’s t-tests and Mann–Whitney U-tests were used prior to the Kolgomorov–Smirnov test to determine normality. Cohen’s d was used to measure effect size. Pearson’s correlation (r) was used to correlate normal data, and Spearman’s correlation coefficient (rs) was used when any of the variables did not follow a normal distribution. A multivariate analysis was performed by binary logistic regression after performing the Hosmer and Lemeshow goodness-of-fit test and using Wald’s backward elimination technique; only the OR (Odds Ratio) with *p* < 0.05 were left in the model.

## 3. Results

Of the 112 preterm infants who participated in the study, according to their weight for gestational age at admission, 25% were of low weight for gestational age, 72.3% were of adequate weight and 2.7% were of high weight. According to diagnoses at admission, 17.9% had respiratory distress, 8% hyaline membrane disease, 1.8% infectious risk and 0.9% transient tachypnoea of the newborn. A total of 56 preterm infants were in the KMC group and 56 in the control group. Demographic and clinical characteristics of the preterm infants and mothers are presented in Table 1. Based on the results of the study, the data were homogeneous in terms of birth weight, anthropometric measurements and Apgar test scores of the preterm infants, as well as demographic and clinical data of the mothers at the time of the intervention, (*p* > 0.05).

At baseline (day 3 of life), mean heart rate, respiratory rate, blood pressure and oxygen saturation were homogeneous in all groups. Mean maximum FiO_2_ concentrations for the intervention and control groups ranged from 21% to 25%, with no statistically significant differences between the two groups. Most participants were maintained on room air or 21% FiO_2_. Similarly, cortisol levels in children and mothers between the groups were homogeneous. EDPS scores in both groups were also similar on the first day of the study.

After the intervention days (15th day), mean cortisol levels in the KMC group were significantly lower than those in the control group in both children (*p* = 0.02) and mothers (*p* = 0.002). Significant changes were also observed in weight gain, quantity, type of feeding and number of feedings, parental nutrition need as well as in the Edinburgh Postpartum Depression Scale (Table 2). Heart rate, respiratory rate, systolic and diastolic blood pressure, oxygen saturation and maximum FiO_2_ administered to the infants were lower in the KMC group compared to the control group, although not statistically significant (*p* > 0.05). The need for parenteral nutrition (TPN) of infants showed a significant difference between KMC (*p* = 0.003) and OR = 0.19 (95% CI, 0.06–0.55), which is a 5.26 times higher risk for the need for TPN in the control group infants than in the KMC group.

As for the total duration in minutes of kangaroo care, the mean total time was 998.57 ± 644.85 min with a minimum of 0 and a maximum of 2880 min (about 48 h).

Pearson’s correlation was performed to see the relationship between the total duration in minutes of kangaroo care and the results of the postintervention variables of the infants and mothers. The result obtained show that there was a negative correlation between the cortisol level of both the infant (r = −0.315; *p* = 0.001) and the mother (r = −0. 216; *p* = 0.02); therefore, the longer the kangaroo care time, the lower the cortisol levels, the greater the weight gain in the infants (r = 0.314; *p* = 0.001) and the higher the number of feedings due to better tolerance (r = 0.295; *p* = 0.002) (Table 3).

A binary logistic regression analysis was applied, after performing the Hosmer and Lemeshow goodness-of-fit test of the model (Chi-squared 5.22; *p* = 0.739), and it was adjusted to a Nagelkerke R-square of 0.12, introducing into the model the variables weeks of gestation, child cortisol, postintervention weight and respiratory frequency and the kangaroo care variable (Yes/No) as the dependent variable. It was observed that increased kangaroo time was a protective factor for cortisol with an OR of 0.88 and that these infants were 3.55 times more likely to gain weight. (Table 4).

## 4. Discussion

In the present study, we examined the effect of kangaroo care and its duration on physiological stress parameters, clinical parameters, cortisol of preterm and maternal infants and EDPS score.

We found significantly lower cortisol levels in preterm infants in the kangaroo group (*p* = 0.02) compared to the control group on the 15th day of life. This finding suggests that at least one benefit of KMC treatment is dependent on the duration of KMC. These patterns of hormonal activity support the interpretation that increased involvement of KMC during the infant’s stay in the NICU may have contributed to the regulation of the adrenocortical function and that the intervention could mitigate some potentially harmful effects of hypercortisolemia [52]. Furthermore, this finding supports the hypothesis that KMC intervention can reduce stress in preterm infants indicating that the mother’s close contact and touch have a buffering effect on infant and maternal stress reactivity. This is also supported by a study by Feldman et al. [53], showing that skin-to-skin contact for one hour a day for the first 14 days of a preterm baby’s life improves the organisation of the baby’s physiological systems, including cortisol reactivity ten years later.

The results obtained show that there was a negative correlation between the total duration of KMC time and both infant and maternal cortisol levels; therefore, the longer the KC time, the lower the cortisol levels. Similar previous results reported that infants who received KC for more than 60 min showed a considerable drop in their cortisol levels [11,54]. These results could indicate that KMC has a relieving effect on the infant’s stress response, which could be explained by the fact that early physical contact with the mother has an impact on the infant’s neuroendocrine pathways that manage stress.

Results from other studies have supported KMC as being a stress-reducing intervention compared to incubator care. Vittner et al. [55] found that salivary cortisol decreased during a 60 min skin-to-skin session; this finding was consistent regardless of whether this was provided by the mother or father (*p* < 0.001). Similarly, Neu et al. [56] found that infant’s salivary cortisol levels decreased during a 1 h skin-to-skin session compared to levels before the session (*p* < 0.01). However, Mirnia et al. found no statistically significant differences in salivary cortisol levels between infants receiving 45 min of SSC with their parents and infants receiving incubator care.

Regarding physiological effects, the results of this study show that the heart rate, respiratory rate, systolic and diastolic blood pressure and oxygen saturation of the infants were lower in the KMC group than in the control group, although they were not statistically significant. However, other studies found that the effect on respiratory rate in preterm infants receiving kangaroo care was significant [18]. Similar results were found by Pados and Hess [22], who investigated whether skin-to-skin contact was an intervention used to reduce stress in the NICU, and the research showed that it produced short-term improvements in cardiorespiratory stress compared to incubator care. Although the effects on these physiological parameters were not significant in the kangaroo intervention, there was no significant increase in these parameters; thus, taken together, these findings suggest that kangaroo therapy contributes to clinical stability. Other studies not only found skin-to-skin contact to be safe for infants with complex congenital heart disease, but also reported improvements in physiological parameters in these particularly vulnerable infants [57]. It also supports our findings as they conclude that the evidence is clearest for studies reporting stress hormone outcomes with strong evidence that KMC reduces cortisol. Ludington-Hoe (2011) [58] reported that kangaroo care affected respiratory rate in preterm infants on the basis that during skin-to-skin care, preterm infants lie with the mothers’ bodies tilted forward 60°, supporting their lung function. Oxygen saturation increased slightly in the group that received more KMC time compared to the control group.

Overall, kangaroo care appears to be beneficial in terms of the oxygen needs of preterm infants, as the upright position may promote the stabilisation of cardiopulmonary function. El-Farrash et al. [11] studied stress response, breastfeeding success and vital signs in preterm infants. After the first KMC session, he observed an improvement in oxygen saturation and temperature in the 120 min KC group compared to the 60 min KC group (*p* < 0.05). Salivary cortisol decreased in both KC groups compared to controls after 7 days (*p* < 0.05).

The effects of KMC over time are significant in terms of weight gain and improved breastfeeding, data that are consistent with the results of other authors such as Wang et al. [28] who studied the effect of the kangaroo method in NICU daily in 2.5 h session. Their results showed that compared to the control group, KMC babies received a higher proportion of breast milk during hospitalisation, lower food intolerance at discharge and a higher proportion of exclusive breastfeeding. Babies cared for in KMC have been shown to have better digestion and metabolism of food and generally develop better and gain weight faster than babies who only receive incubator care [59,60]. Most studies reported that the weight of babies who received KMC increased, but it is also difficult to control for other unavoidable variables in the NICU, such as intravenous fluids and treatment.

It is of utmost importance to find methods of care that can buffer stress and thus long-term elevated cortisol levels in preterm infants in the NICU. These findings suggest that KMC may have substantial benefits for maternal mental health, results similar to the recent study by Sinha et al. [61] who estimated the effects of community-initiated KMC on maternal risk of moderate to severe postpartum depressive symptoms and on salivary cortisol concentration. KMC can be used as a nonpharmacological intervention to prevent or decrease the risk of postpartum depression. Mothers of very preterm infants are at greater risk of experiencing stress and depression after birth than mothers of full-term infants [62].

Unfamiliarity with the NICU environment, the fragile appearance of the preterm infant, disruption in caregiving roles and constant worry that the baby will die are well recognised causes of stress for mothers of preterm infants [63]. The results of our study show a significant decrease in maternal cortisol levels as well as in the postpartum depression scale. Similar results were found by Vittner et al. [55] who examined the changes occurring in infant and parent salivary cortisol levels during skin-to-skin contact and whether this intervention relieved parental stress and anxiety, and the results showed that infant salivary cortisol levels decreased significantly (*p* < 0.001) during skin-to-skin contact, and parent anxiety scores were significantly related to parent cortisol levels. Cong et al. [64] examined the mechanism of cortisol in modulating parental stress and anxiety during skin-to-skin with their preterm infants, finding that maternal cortisol continuously decreased after this intervention. Morelius et al. [65], concluded that skin-to-skin contact reduces infant cortisol reactivity in response to treatment and improves the concordance between maternal and infant salivary cortisol levels. Other studies also support the findings that kangaroo care can be an effective intervention to reduce parental and infant stress in the NICU [21,66], as well as decreasing anxiety and improving symptoms of postpartum depression and general aspects of mood [67]. Mothers play an important role in infant care, and positive interactions between mothers and preterm infants at an early age have been shown to be associated with better cognitive outcomes [32,34] suggesting maternal involvement constitutes a very positive intervention [35].

In hospitals around the world, the practice of KMC has recently begun to gain more attention and importance in relation to neurodevelopmentally focused care of preterm infants [68] and in improving family-centred care in hospitals [69].

### 4.1. Limitations

With the sample size of this study, statistically significant results were obtained with positive effects showing that KMC decreases the cortisol levels of infants and mothers; however, research with a larger sample size would be appropriate to improve the population generalisability of these results. Further, the study was limited to 12 days of data collection; a longer duration of the kangaroo method may be able to confirm whether the decrease in and/or stabilisation of physiological stress parameters is progressive over time.

In addition, future work should consider collecting data on variables that might influence the results, such as the average number of painful procedures per day experienced by preterm infants or the severity of their illness. Recent studies show that during the first weeks in the NICU, preterm infants experience a daily mean of 22.97 ± 2.30 stressful procedures and a mean of 42.59 ± 15.02 h of cumulative times of chronic/stressful exposure [6]. Despite being a public hospital, where all the women in the study were covered by the Spanish social security system, which means that they are entitled to the corresponding maternity leave and could therefore perform kangaroo care without work-related impediments, we do consider that some mothers may have had certain difficulties in carrying out this method due to living far from the hospital, needing transport or having to care for other children. Moreover, in hospitals around the world, the practice of KMC has recently begun to gain more attention and importance; however, concerns about the transmission of Severe Acute Respiratory Syndrome Coronavirus-2 (SARS-CoV-2), the new 2019 coronavirus outbreak (COVID-19) and the subsequent COVID-19-related restrictions on hospital procedures, could raise the level of prenatal psychological distress for both women and their babies [21].

### 4.2. Implication for Practitioners and Researchers

The practice of kangaroo care continues to evolve and progress within facilities caring for mothers and newborns born preterm. Kangaroo practices for preterm infants in the NICU provide physiological stability. In addition, the mother benefits from reduced stress, improved breastfeeding outcomes and more positive attachment behaviour. Kangaroo care benefits the baby by providing a buffer from the physical environment of the NICU, as well as helping to regulate following subsequent environmental disruption. Providing parents with a supportive environment in the NICU can facilitate active involvement in the care of the infant, which is of significant benefit to the developing infant. Neonatal intensive care providers have the ability and responsibility to influence the practice of kangaroo care as well as to value kangaroo care as a beneficial practice. Promoting education and seeking involvement within the healthcare team can help ensure implementation of this evidence-based practice that has the potential to improve health outcomes for preterm infants and their mothers.

Our results help health professionals and mothers of preterm infants by showing that at least 90 min of kangaroo care per day during the first two weeks of life improves their infant’s weight gain, decreases their stress and improves their physiological constants, of particular interest in underdeveloped or developing countries. The findings advance the exploration of serum cortisol as a potential marker to assess the responsiveness of preterm infants to stress and improve synchrony in mother–infant interactions.

## 5. Conclusions

KMC for more than 90 min daily in preterm infants during the first 2 weeks of life statistically significantly decreases preterm and maternal blood cortisol levels and improves weight gain and contributes to improved physiological constants of the infants as well as mitigating maternal postpartum depression. Our results support our hypothesis that the practice of early KMC intervention in the NICU reduces stress in both preterm infants and mothers and promotes the health and wellbeing of preterm infants. The greater the close and direct contact of mothers with preterm infants, the greater the buffering effect is on infant and maternal stress. Further studies are required to assess the optimal duration of KMC to optimise the beneficial effects in preterm infants, as well as in other infants with other medical complexities who may benefit significantly from this intervention.

## Figures and Tables

**Table 1:** Description of Infant and Maternal Clinical and Demographic Characteristics of KMC and control groups.

Variable		KMC Group (*n* = 56 ) *n* (%), Mean ± SD	Control Group (*n* = 56 ) *n* (%), Mean ± SD	d.f.	Test	*p*-Value	Cohen’s d
***Premature Infant Variables***							
Gender	Male	28 (25)	36 (32.14)	1	χ^2^ = 2.33	0.12 _a_	--
	Female	28 (25)	20 (17.85)				
Gestational age (weeks)		31.12 ± 1.81	30.38 ± 1.98	110	t = 2.05	0.043 _B_ *	0.39
Weight at birth (g)		1408.86 ± 301.67	1327.61 ± 292.02	110	t = 1.44	0.15 _B_	0.27
Length at birth (cm)		39.85 ± 3.67	38.91 ± 3	110	t = 1.48	0.14 _B_	0.55
Head circumference at birth (cm)		28.33 ± 2.35	27.77 ± 2.15	--	U = 1305	0.12 _A_	0.24
Chest circumference at birth (cm)		24.54 ± 2.19	24.77 ± 1.82	--	U = 1529	0.82 _A_	0.11
Apgar at 1 min		9.95 ± 1.93	7.38 ± 2.05	--	U = 1263	0.08 _A_	1.29
Apgar at 5 min		9.54 ± 1.54	9.23 ± 0.87	--	U = 1371	0.20 _A_	0.24
***Mother Variables***							
Mother age (year)		32.84 ± 5.64	33.16 ± 5.32	--	U = 1490	0.65 _A_	0.05
Previous pregnancies		1.45 ± 1.48	1.14 ± 1.34	--	U = 1383	0.26 _A_	0.21
Previous abortions		0.61 ± 0.98	0.55 ± 0.93	--	U = 1489	0.59 _A_	0.06
Multiple birth	No	38 (33.92)	36 (32.14)	1	χ^2^ = 0.159	0.69 _a_	--
	Yes	18 (16.07)	20 (8.92)				
Smoker	No	47 (42.00)	48 (42.90)	1	χ^2^ = 0.069	0.79 _a_	--
	Yes	9 (8.00)	8 (7.10)				
No. of cigarettes		0.52 ± 4.09	0.52 ± 4.09	--	U = 1557	0.92 _A_	0

_A_ Mann–Whitney U; _B_ Student’s *t*-test; _a_ Chi-squared; d.f.: degrees of freedom; * *p* < 0.05.

**Table 2:** Effects of Kangaroo Mother Care on Physiological Parameters (N = 112).

			Day 3					Day 15					
***Premature Infant: Quantitative Variables***													
**Variable**		**Group**	**Mean**	**SD**	**d.f**	**Test**	***p*-Value**	**Mean**	**S.D.**	**d.f**	**Test**	***p*-Value**	**OR (95% CI)**
Cortisol Infant (µg/dL)		**KMC**	9.51	5.42	110	0.4	0.86 _B_	4.16	2.82	110	2.39	0.02 _B_ *	--
		**Control**	10.03	6.11				5.89	4.61				
Heart rate (beats/min)		**KMC**	150.38	15.10	110	0.55	0.50 _B_	154.29	13.67	110	1.79	0.11 _B_	--
		**Control**	151.93	14.31				158.43	10.61				
Respiratory rate (breaths/min)		**KMC**	52.54	15.07	110	0.84	0.34 _B_	52.89	10.55	110	2.07	0.05 _B_	--
		**Control**	54.68	15.07				59.25	20.36				
Systolic blood pressure (mm Hg)		**KMC**	67.70	11.04	110	0.67	0.75 _B_	69.77	10.80	110	1.05	0.40 _B_	--
		**Control**	69.21	12.75				72.11	12.52				
Diastolic blood pressure (mm Hg)		**KMC**	40.27	10.15	110	−0.34	0.43 _B_	39.07	11.07	110	−0.15	0.72 _B_	--
		**Control**	39.55	11.73				38.77	9.40				
O_2_ saturation (%)		**KMC**	97.39	1.69	110	−1.55	0.31 _B_	97.30	8.15	110	1.22	0.86 _B_	--
		**Control**	94.89	11.92				95.93	8.15				
Max FiO_2_		**KMC**	21.77	2.79	110	1.733	0.23 _B_	21.03	4.56	110	−0.18	0.42 _B_	--
		**Control**	23.10	5.04				21.17	3.62				
Weight		**KMC**	1329.90	265.96	--	1237	0.06 _A_	1567.20	287.02	--	1171	0.01 _A_ *	--
		**Control**	1239.61	245.04				1440.59	277.64				
Amount per feeding		**KMC**	10.13	8.18	110	−0.44	0.44 _B_	32.74	8.5	110	−2.64	0.01 _B_ *	--
		**Control**	9.41	8.88				26.58	15.55				
Number of feedings		**KMC**	7.86	1.06	110	−1.95	0.06 _B_	8.00	0.00	110	−1.83	0.04 _B_ *	--
		**Control**	7.16	2.44				7.55	1.81				
Number of puffs		**KMC**	0.38	0.98	110	0.45	0.83 _B_	0.04	0.18	110	1.16	0.61 _B_	--
		**Control**	0.32	0.69				00.13	0.54				
***Premature Infant Qualitatives Variables***													
**Variable**		**Group**	***n***	**%**	**d.f**	**Test**	***p*-Value**	***n***	**%**	**d.f**	**Test**	***p*-Value**	**OR (95% CI)**
Respiratory therapy	Ambiental	**KMC**	37	33.03	5	10.49	0.06 _b_	49	43.75	3	9.54	0.02 _b_ *	--
		**Control**	25	22.32				39	34.82				
	Oxygen incubator	**KMC**	0	0				1	0.89				
		**Control**	3	2.67				3	2.67				
	Nasal goggles	**KMC**	1	0.89				0	0				
		**Control**	1	0.89				0	0				
	High flow	**KMC**	3	2.67				5	4.46				
		**Control**	5	4.46				5	4.46				
	Noninvasive ventilation	**KMC**	15	13.39				1	0.89				
		**Control**	20	17.85				9	8.03				
	Endotracheal intubation	**KMC**	0	0				0	0				
		**Control**	2	1.78				0	0				
Parenteral nutrition	No	**KMC**	14	12.5	1	0.18	0.67 _a_	51	45.53	1	8.96	0.003 _c_ *	0.19 (0.06–0.55)
		**Control**	16	14.28				37	33.03				
	Yes	**KMC**	42	37.05				5	4.46				
		**Control**	40	35.71				19	16.96				
Type of milk	Breast	**KMC**	48	42.85	2	7.99	0.01 _b_ *	54	48.21	2	7.42	0.02 _b_ *	--
		**Control**	50	44.64				50	44.64				
	Mixed	**KMC**	5	4.46				2	1.78				
		**Control**	0	0				1	0.89				
	Artificial	**KMC**	3	2.67				0	0				
		**Control**	6	5.35				5	4.46				
Type of feeding	Absolute diet	**KMC**	1	0.89	2	4.53	0.10 _b_	0	0	3	6.48	0.09 _b_	--
		**Control**	5	4.46				3	2.67				
	Intermittent	**KMC**	55	49.10				51	45.53				
		**Control**	50	44.64				46	41.07				
	Continuous	**KMC**	0	0				1	0.89				
		**Control**	1	0.89				4	3.57				
	Bottle	**KMC**	0	0				4	3.57				
		**Control**	0	0				3	2.67				
***Mother: Quantitative Variables***													
**Variable**		**Group**	**Mean**	**SD**	**d.f**	**Test**	***p*-Value**	**Mean**	**S.D.**	**d.f**	**Test**	***p*-Value**	**OR (95% CI)**
Mother Cortisol(µg/dL)		**KMC**	8.27	5.04	--	1472.50	0.81 _A_	5.87	3.35	--	1038.00	0.002 _A_ *	--
		**Control**	8.58	4.75				7.65	3.81				
EPDS		**KMC**	17.28	2.74	--	1534.00	0.81 _A_	15.39	2.40	--	1177.50	0.002 _A_ *	--
		**Control**	17.62	2.55				16.67	2.82				

Note. KMC group (*n*  = 56); Control group (*n*  = 56); _A_ Mann–Whitney U; _B_ Student’s *t*-test; _a_ Chi-squared; _b_ Likelihood ratio; _c_ Yates correction; d.f.: degrees of freedom; * *p* < 0.05.

**Table 3:** Pearson’s correlation between the total duration (min.) of KMC and the outcomes of infant and maternal postintervention variables.

Postintervention	N	Pearson’s Correlation Coefficient	*p*-Value
***Premature Infant Variables***			
Cortisol Infant	112	−0.315 _b_	0.001
Heart rate (beats/min)	112	−0.153	0.10
Respiratory rate (breaths/min)	112	−0.164	0.08
Systolic blood pressure (mm Hg)	112	−0.094	0.32
Diastolic blood pressure (mm Hg)	112	−0.018	0.85
O_2_ saturation (%)	112	−0.103	0.28
Max FiO_2_	112	0.017	0.85
Weight	112	0.314 _b_	0.001
Amount per feeding	112	0.295 _b_	0.002
Number of feedings	112	0.219 _a_	0.20
Number of puffs	112	−0.035	0.71
***Mother Variables***			
Mother Cortisol	112	−0.216 _a_	0.02
EPDS	112	−0.236	0.01

_a_ Low correlation; _b_ moderate to weak correlation.

**Table 4:** Binary logistic regression analysis of kangaroo mother method performance.

Variable Day 15	ORa	95% C.I.	*p*-Value
Cortisol Infant	0.88	0.77–0.99	0.043
Weight	3.55	1.89–6.67	0.041
